# Biomarkers of Histone Deacetylase Inhibitor Activity in a Phase 1 Combined-Modality Study with Radiotherapy

**DOI:** 10.1371/journal.pone.0089750

**Published:** 2014-02-25

**Authors:** Anne Hansen Ree, Marie Grøn Saelen, Erta Kalanxhi, Ingrid H. G. Østensen, Kristina Schee, Kathrine Røe, Torveig Weum Abrahamsen, Svein Dueland, Kjersti Flatmark

**Affiliations:** 1 Department of Oncology, Akershus University Hospital, Lørenskog, Norway; 2 Institute of Clinical Medicine, University of Oslo, Oslo, Norway; 3 Department of Tumor Biology, Oslo University Hospital – Norwegian Radium Hospital, Oslo, Norway; 4 Department of Genes and Environment, Norwegian Institute of Public Health, Oslo, Norway; 5 Department of Oncology, Oslo University Hospital – Norwegian Radium Hospital, Oslo, Norway; 6 Department of Gastroenterological Surgery, Oslo University Hospital – Norwegian Radium Hospital, Oslo, Norway; Texas A&M University, United States of America

## Abstract

**Background:**

Following the demonstration that histone deacetylase inhibitors enhanced experimental radiation-induced clonogenic suppression, the Pelvic Radiation and Vorinostat (PRAVO) phase 1 study, combining fractionated radiotherapy with daily vorinostat for pelvic carcinoma, was designed to evaluate both clinical and novel biomarker endpoints, the latter relating to pharmacodynamic indicators of vorinostat action in clinical radiotherapy.

**Patients and Methods:**

Potential biomarkers of vorinostat radiosensitizing action, not simultaneously manifesting molecular perturbations elicited by the radiation itself, were explored by gene expression array analysis of study patients' peripheral blood mononuclear cells (PBMC), sampled at baseline (T0) and on-treatment two and 24 hours (T2 and T24) after the patients had received vorinostat.

**Results:**

This strategy revealed 1,600 array probes that were common for the comparisons T2 *versus* T0 and T24 *versus* T2 across all of the patients, and furthermore, that no significantly differential expression was observed between the T0 and T24 groups. Functional annotation analysis of the array data showed that a significant number of identified genes were implicated in gene regulation, the cell cycle, and chromatin biology. Gene expression was validated both in patients' PBMC and in vorinostat-treated human carcinoma xenograft models, and transient repression of *MYC* was consistently observed.

**Conclusion:**

Within the design of the PRAVO study, all of the identified genes showed rapid and transient induction or repression and therefore, in principle, fulfilled the requirement of being pharmacodynamic biomarkers of vorinostat action in fractionated radiotherapy, possibly underscoring the role of *MYC* in this therapeutic setting.

## Introduction

Modern radiation oncology will require a synergy between high-precision radiotherapy protocols and innovative approaches for biological optimization of radiation effect. From a clinical perspective, new insights into molecular radiobiology will provide a unique opportunity for combining systemic targeted therapeutics with radiotherapy [1]. One example is the use of histone deacetylase (HDAC) inhibitors as potentially radiosensitizing drugs. Inhibition of HDAC enzymes leads to acetylation of histone and non-histone proteins, and the resultant changes in gene transcription cause alterations in key molecules that orchestrate a wide range of cellular functions, including cell cycle progression, DNA damage signaling and repair, and cell death by apoptosis and autophagy [2]–[5].

Following the demonstration that HDAC inhibitors enhanced radiation-induced clonogenic suppression of experimental *in vitro* and *in vivo* colorectal carcinoma models [6]–[9], but independently of the actual histone acetylation level at the time of radiation exposure [7], [8], we conducted the Pelvic Radiation and Vorinostat (PRAVO) phase 1 study [10], [11]. This trial, undertaken in sequential patient cohorts exposed to escalating dose levels of the HDAC inhibitor vorinostat combined with pelvic palliative radiotherapy for advanced gastrointestinal malignancy, was the first to report on the therapeutic use of an HDAC inhibitor in clinical radiotherapy. It was designed to demonstrate a number of key questions; whether the investigational agent reached the specific target (detection of tumor histone acetylation), the applicability of non-invasive tumor response assessment (using functional imaging), and importantly, that the combination of an HDAC inhibitor and radiation was safe and tolerable.

The ultimate goal of a first-in-human therapy trial is to conclude with a recommended treatment dose for follow-up expanded trials, and in achieving this, a phase 1 study typically is designed to determine treatment toxicity and tolerability (in terms of dose-limiting toxicity and maximum-tolerated dose (MTD), respectively) [12], [13]. For molecularly targeted agents, the dose that results in a relevant level of target modulation may differ greatly from the MTD, and generally, we do not have a good understanding of the relationship between the MTD and the dose required to achieve the desired therapeutic effect [1]. An optimum biological dose may be the dose that is associated with pharmacodynamic biomarkers reflecting the mechanism of drug action. In the setting of fractionated radiotherapy, this would ideally represent a radiosensitizing molecular event occurring at each radiation fraction, or in other words, a biological indicator with a transient and periodic expression profile. Importantly, tumor specimens for this particular purpose cannot be sampled after the patient has commenced the radiation treatment. Any signaling activity in on-treatment tumor samples would reflect the combined effect of radiation and the systemic drug, and the contribution of the latter would probably be indistinguishable from the effect of the actual accumulated radiation dose. Instead, the study can be designed to collect non-irradiated surrogate tissue both before the commencement of study treatment and on-treatment at time points reflecting the timing of administration of the systemic drug with regard to the fractionated radiotherapy protocol. In addition, as a general rule, biomarkers that have been previously established for single-agent therapy will require reevaluation in a first-in-human clinical trial combining a molecularly targeted compound with radiotherapy.

Within this context, *i.e.,* that the possible mechanism of radiosensitizing action of the molecularly targeted agent should be regarded a main objective in a combined-modality study with radiotherapy, the present study reports on a correlative analytical strategy for identifying possible biomarkers of HDAC inhibitor activity, using peripheral blood mononuclear cells (PBMC) from the PRAVO phase 1 study patients receiving pelvic palliative radiotherapy as an easily accessible surrogate tissue for vorinostat exposure [14]. Gene expression array analysis identified PBMC genes that from experimental models are known to be implicated in biological processes governed by HDAC inhibitors, and might be further developed as pharmacodynamic biomarkers of vorinostat activity in the setting of fractionated radiotherapy.

## Materials and Methods

### Ethics Statement

Both of the protocols for the PRAVO study (ClinicalTrials ID NCT00455351) and the phase 2, non-randomized study for patients with locally advanced rectal cancer (LARC) given neoadjuvant chemoradiotherapy (ClinicalTrials ID NCT00278694) were approved by the Institutional Review Board and the Regional Committee for Medical and Health Research Ethics South-East Norway (REC South-East, Permit Number S-06289 and S-05059, respectively), and were performed in accordance with the Declaration of Helsinki. Written informed consent was required for participation. Housing and all procedures involving animals were performed according to protocols approved by the Animal Care and Use Committee at Department of Comparative Medicine, Oslo University Hospital (Permit Number 885–2616–2919–2928–3688), in compliance with the Norwegian National Committee for Animal Experiments' guidelines on animal welfare.

### PRAVO Study Patients and Objectives

The patient population was enrolled between February 2007 and May 2009. The principal eligibility criterion was histologically confirmed pelvic carcinoma scheduled to receive palliative radiation to 30 Gy in 3-Gy daily fractions. Other details on eligibility are given in the initial report [10]. This phase 1 dose-escalation study adopted a standard 3+3 expansion cohort design [12], where patients with advanced gastrointestinal carcinoma were enrolled onto four sequential dose levels of vorinostat (Merck & Co., Inc., Whitehouse Station, NJ, USA), starting at 100 mg daily with dose escalation in increments of 100 mg [10]. The primary objective was to determine tolerability of vorinostat, defined by dose-limiting toxicity and MTD, when administered concomitantly with palliative radiation to pelvic target volumes. Secondary objectives were to assess the biological activity of vorinostat, including the identification of possible biomarkers of HDAC inhibitor activity, and to monitor radiological response when given with pelvic radiotherapy. The study data on patient treatment tolerability, tumor histone acetylation following vorinostat administration, and treatment-induced changes in tumor volume and apparent distribution coefficient, as assessed by magnetic resonance imaging, have been reported in detail previously [10], [11].

### Patient Blood Sampling and RNA Isolation

**Figure 1:**
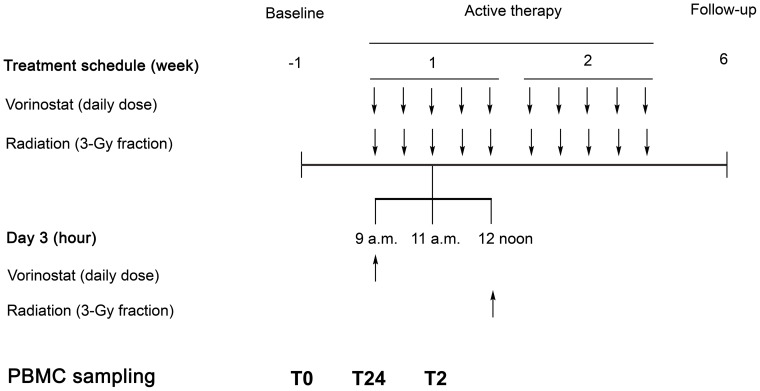
Treatment schedule for the Pelvic Radiation and Vorinostat phase 1 study. This study combined pelvic palliative radiotherapy (30 Gy in 3-Gy daily fractions; administered at 12 noon) with the histone deacetylase inhibitor vorinostat (given once daily at 9 a.m.) for advanced gastrointestinal malignancy. Arrows indicate administration of therapy. Study patients' peripheral blood mononuclear cells (PBMC) were sampled before commencement of treatment (T0) and on active therapy, two hours (T2; at 11 a.m. on day 3) and 24 hours (T24; at 9 a.m. on day 3) after the previous dose of vorinostat.

As depicted by Figure 1, peripheral blood, drawn on PAXgene Blood RNA Tubes (Qiagen Norge, Oslo, Norway), was collected at baseline (before commencement of study treatment; termed T0) and on-treatment the third treatment day, two and 24 hours after the patient had received the preceding daily dose of vorinostat (termed T2 and T24), respectively. A full set of three samples (T0, T2, and T24) was obtained from 14 of the 16 evaluable study patients (Table 1). The tubes were stored at −70°C until analysis. Total PBMC RNA was isolated using PAXgene Blood RNA Kit (Qiagen), following the manufacturer's protocol. RNA concentration and quality were assessed using NanoDrop 1000 and Agilent 2100 Bioanalyzer (Thermo Fisher Scientific Norway, Oslo, Norway), respectively.

**Table 1:** Study patients.

Vorinostat dose (mg daily)	Age (years)	Gender	Comment
100	77	female	
200	49	female	
200	64	female	
200	66	female	
300	47	female	PBMC a not available
300	66	female	
300	77	male	
300	81	female	
300	82	male	
300	87	female	PBMC a not available
400	45	female	
400	55	male	
400	62	male	
400	75	female	
400	83	female	
400	85	female	

a Peripheral blood mononuclear cells.

### Gene Expression Array Analysis

This analysis was performed by the Norwegian Genomics Consortium (Oslo, Norway). Briefly, cRNA synthesis, amplification, and hybridization to Illumina Human WG-6 v3 Expression BeadChip arrays (Illumina, Inc., San Diego, CA, USA), containing 48,000 probes, were carried out as per manufacturer's instructions. Signal intensities were extracted by the BeadArray Reader Software (Illumina), and raw data were imported into the GenomeStudio v2010.1 Software, Gene Expression module v1.6.0 (Illumina). The primary array data are available in the Gene Expression Omnibus data repository (GEO accession number GSE46703).

### Statistical and Functional Annotation Analyses of Array Data

Analysis was performed using Bioconductor vR2.11.1 and the Bioconductor packages lumi 1.14.0, linear models for microarray data (limma) 3.4.4, and illuminaHumanv3BeadID.db 1.6.0 (www.bioconductor.org). Following quality control and pre-processing, the data were log_2_-transformed, and differential gene expression between the sample groups T0, T2, and T24 was determined by applying a Benjamin and Hochberg false discovery rate-adjusted *P*-value cut-off of 0.05. The total number of probes that were identified as differentially expressed was analyzed using the Database for Annotation, Visualization and Integrated Discovery, DAVID v6.7 [15], [16]. Enriched biological processes and pathways were identified using the GOTERM_BP_FAT and KEGG_PATHWAY algorithms, applying a *P*-value cut-off of 0.01. Differential expression analysis of the array data was also performed using a *P*-value of 0.01 and a log_2_-fold change cut-off of 1.0 in order to identify genes whose expression changes could have potentially high biological significance.

### Experimental Human Colorectal Carcinoma Models

The HCT116 and SW620 colorectal carcinoma cell lines were originally purchased from American Type Culture Collection (Manassas, VA, USA), and the identities of our laboratory's versions were confirmed by short tandem repeat analysis (Table S1). The LoVo-92 colorectal carcinoma cell line was kindly provided by Dr. Paul Noordhuis (VU Medical Centre, Amsterdam, The Netherlands) [17]. The cell lines were cultured as previously described [8], [17]. Xenografts were established by subcutaneous injections of HCT116 or SW620 cell suspensions (2×10^6^ cells) bilaterally on the flanks of locally bred female BALB/c nude (nu/nu) or Athymic Nude-Foxn1^nu^ mice, 6–8 weeks of age. Vorinostat (Cayman Chemical, Ann Arbor, MI, USA; 100 mg/kg, dissolved in dimethyl sulfoxide to a concentration of 100 mg/ml immediately before use) or vehicle was given by intraperitoneal injection 13 days (HCT116) or 20 days (SW620) after establishment of xenografts. Three and 12 hours after administration, the tumors were extirpated, snap-frozen in liquid nitrogen, and stored at −70°C. The xenografts were sectioned using a cryostat microtome prior to RNA extraction using TRIzol® Reagent (Invitrogen Dynal AS, Oslo, Norway). RNA concentration was assessed using the RNA/DNA calculator Gene Quent II (Pharmacia Biotech, Piscataway, NJ, USA).

### Tumor Samples from LARC Patients

Primary tumor biopsies were sampled at the time of diagnosis from LARC patients enrolled onto a phase 2 study on neoadjuvant chemoradiotherapy (Table S2). The biopsy samples were snap-frozen in liquid nitrogen and stored at −70°C, and sectioned on the cryostat microtome, essentially as previously reported [18], before RNA was extracted.

### Reverse Transcriptase Quantitative Polymerase Chain Reaction (RT-qPCR) Analysis

cDNA was synthesized from total RNA using the qScript™ cDNA Synthesis Kit (Quanta BioSciences, Inc., Gaithersburg, MD, USA). The qPCR was run in Perfecta qPCR Supermix (Quanta), on iCycler (Bio-Rad Laboratories Norway, Oslo, Norway) and with all reactions in parallel. Primers were designed using ProbeFinder Assay Design Software (www.roche-applied-science.com/sis/rtpcr/upl/ezhome.html), and were obtained from the Universal ProbeLibrary collection (Roche Applied Sciences, Oslo, Norway). Primer sequences are listed in Table S3. Amplified cDNA generated from the reference cell line (LoVo-92) was included on all PCR plates for relative quantification purposes (correction of plate-to-plate variation). Data were normalized to the expression levels of two reference genes; *YARS*, encoding tyrosyl-tRNA synthetase, and *TBP*, encoding the TATA box-binding protein. When tested in the patient samples, the reference genes had equal expression per ng of cDNA, independent of patient treatment (vorinostat dose and time after administration). The data were analyzed using the GeneExpression Analysis for iCycler iQ® Real-Time PCR Detection System Software (BioRad), and were calculated relative to the level in the reference cell line and subsequently log_2_-transformed.

### Statistical Analysis of qPCR Data

Analysis was performed using Predictive Analytics SoftWare Statistics version 19.0 (SPSS Inc., Chicago, IL, USA). Q-Q plots were applied to test whether the data were normally distributed or not, before differences between groups were analyzed using two-sided Student *t*-test for the PBMC samples and Mann-Whitney *U* test for xenograft samples. *P*-values less than 0.05 were considered statistically significant.

## Results

### PBMC Transcriptional Response to Vorinostat – Biological Processes and Pathways

**Figure 2:**
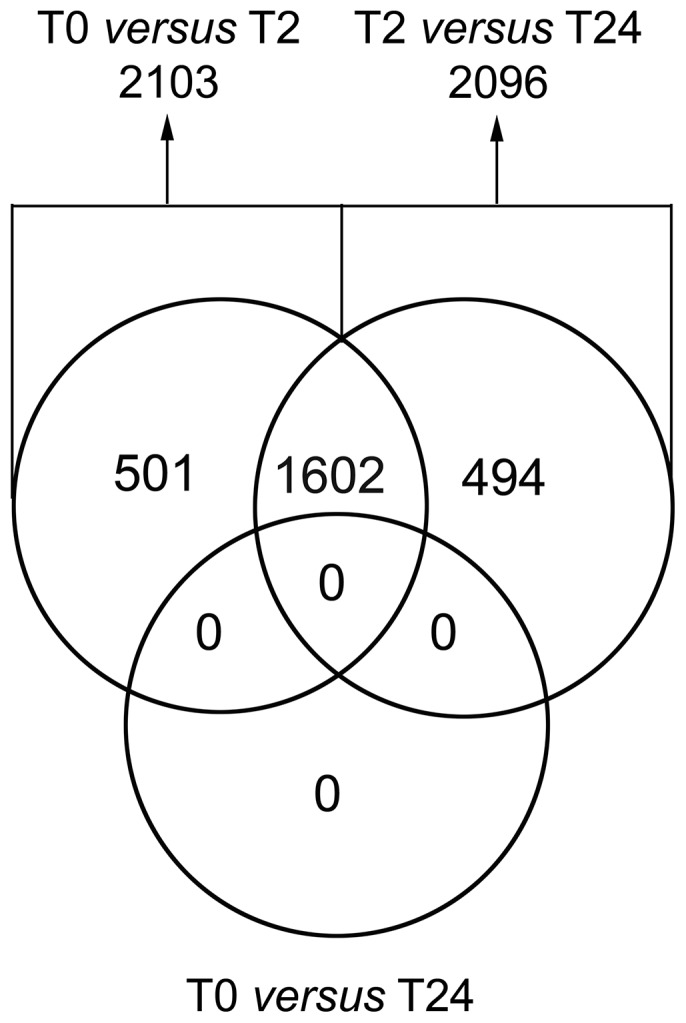
Venn diagram illustrating differentially expressed genes. Study patients' peripheral blood mononuclear cells were sampled at baseline (T0) and on-treatment two and 24 hours after administration of the daily dose of the study medication vorinostat (T2 and T24, respectively). Gene expression was analyzed by Illumina Human WG-6 v3 Expression BeadChip arrays. The array data from all patient samples at each time point (T0, T2, and T24) were pooled for the analysis. Probes with false discovery rate-adjusted *P*-values less than 0.05 were considered differentially expressed and subjected to Venn analysis, comparing by pairs T2 *versus* T0, T24 *versus* T2, and T24 *versus* T0. The figures represent numbers of probes in common for the various conditions.

Table 1 gives study patient baseline characteristics; the full study data on treatment tolerability and response have been reported previously [10], [11]. Of the 14 patients that provided a full set of PBMC samples (T0, T2, and T24), one patient was treated at vorinostat 100 mg once daily and three patients at the 200 mg dose level, whereas four and six patients received the medication at 300 or 400 mg once daily, respectively. Importantly, as vorinostat-induced tumor histone acetylation had been observed at all dose levels [10], the array data from all patient samples at each time point (T0, T2, and T24) were pooled, irrespective of the vorinostat dose administered to the patients. This was done to increase the statistical power of the testing on analysis of differential gene expression between the individual time points. As shown by Figure 2, approximately 2,100 probes were differentially expressed both at two hours of vorinostat exposure (T2 *versus* T0) and on the T24 *versus* T2 comparison when applying the *P*-value cut-off of 0.05. Of these, 1,602 transcripts were found to be altered in both comparisons, and furthermore, no significantly differential expression was observed when comparing the T0 and T24 groups. Hence, all of the 1,602 mutual probes that were identified had a transient change in expression level from T0, with approximately one half found to be up-regulated and thus, the other half down-regulated at T2, followed by the opposite directional change to baseline expression at T24 (data not shown).

Functional annotation analysis of the differentially expressed genes in patients' PBMC identified several enriched biological processes. Comparison of the baseline PBMC transcription profile with that obtained two hours after vorinostat administration (T2 *versus* T0) showed that 69 biological processes were over-represented, whereas the corresponding comparison of T24 *versus* T2 transcriptional profiles identified 106 processes (Table S4). As seen from Table 2, displaying the top-ten Gene Ontology terms for each of the two comparisons, seven out of the ten biological processes were present in both, with transcription being the most significant. In addition, the analysis identified enrichment of genes involved in catabolic processes, the cell cycle, RNA processing, chromatin modification, and chromosome organization. The top-three pathway networks for each of the two comparisons, in common for both, comprised signaling factors of the cell cycle, including the p53 pathway (Table 3).

**Table 2:** Enriched biological processes in patients' peripheral blood mononuclear cells during 24 hours of vorinostat treatment.

Biological process a	*n* (%)	*P*-value	Selected transcripts b
T2 *versus* T0 c			
**GO:0006350** transcription	253 (17)	5.1×10^−14^	*MYC*, *DDIT3*
**GO:0044265** cellular macromolecule catabolic process	107 (7.2)	8.2×10^−11^	*MYC*, *BARD1*
**GO:0044257** cellular protein catabolic process	93 (6.3)	1.7×10^−10^	*BARD1*
**GO:0007049** cell cycle	111 (7.5)	2.3×10^−10^	*MYC*, *MSH6*, *BARD1*, *DDIT3*
GO:0051603 proteolysis involved in cellular protein catabolic process	92 (6.2)	2.9×10^−10^	*BARD1*
GO:0019941 modification-dependent protein catabolic process	89 (6.0)	3.4×10^−10^	*BARD1*
**GO:0009057** macromolecule catabolic process	111 (7.5)	3.4×10^−10^	*MYC, BARD1*
GO:0030163 protein catabolic process	94 (6.6)	3.9×10^−10^	*BARD1*
**GO:0006396** RNA processing	84 (5.6)	1.8×10^−9^	
**GO:0045449** regulation of transcription	276 (19)	5.0×10^−9^	*MYC, DDIT3*
T24 *versus* T2 c			
**GO:0006350** transcription	260 (17)	8.3×10^−16^	*MYC*, *DDIT3*
**GO:0007049** cell cycle	114 (7.5)	2.6×10^−11^	*MYC*, *MSH6*, *BARD1*, *DDIT3*
**GO:0045449** regulation of transcription	286 (19)	5.4×10^−11^	*MYC, DDIT3*
GO:0016568 chromatin modification	55 (3.6)	1.3×10^−10^	
**GO:0006396** RNA processing	86 (5.6)	3.7×10^−10^	
**GO:0044265** cellular macromolecule catabolic process	104 (6.8)	8.8×10^−10^	*MYC*, *BARD1*
GO:0051276 chromosome organization	78 (5.1)	9.6×10^−10^	*MSH6*
GO:0022402 cell cycle process	85 (5.6)	4.1×10^−9^	*MYC*, *MSH6*, *BARD1*, *DDIT3*
**GO:0044257** cellular protein catabolic process	89 (5.8)	4.3×10^−9^	*BARD1*
**GO:0009057** macromolecule catabolic process	107 (7.0)	6.4×10^−9^	*MYC*, *BARD1*

a Gene Ontology (GO) terms in bold: present in both comparisons.

b Verified by reverse transcriptase quantitative polymerase chain reaction analysis.

c T0 represents baseline peripheral blood mononuclear cells (PBMC) samples; T2 and T24 represent PBMC samples collected two and 24 hours, respectively, after the patients had received the daily dose of vorinostat.

**Table 3:** Enriched biological pathways in patients' peripheral blood mononuclear cells during 24 hours of vorinostat treatment.

Biological pathway	*n* (%)	*P*-value	Genes a
hsa04130 SNARE interactions in vesicular transport	10 (0.85)	1.6×10^−4^	*STX6, STX5, STX1A, STX12, STX16, USE1, BET1, BET1L, GOSR1, VAMP1*
hsa04115 p53 signaling pathway	13 (1.1)	2.7×10^−4^	*PMAIP1, RRM2B, SESN2, CDK4, CDK2, CCNE2, CCNE1, PPM1D, TNFRSF10B, RCHY1, APAF1, * ***GADD45B*** *, GADD45A*
hsa04110 cell cycle	17 (1.5)	0.0012	*CCNH, ANAPC13, CDC23, CDK7, PTTG1, CDK4, ZBTB17, TGFB1, WEE1, CDK2, CCNE2, CCNE1, YWHAG, CDKN2D, * ***GADD45B*** *, GADD45A, * ***MYC***

a Genes in bold: verified by reverse transcriptase quantitative polymerase chain reaction analysis.

### Vorinostat Activity in PBMC – Verification of Selected Biomarkers

Next, by introducing a log_2_-fold change cut-off of 1.0 while decreasing the *P*-value to 0.01 in order to identify gene expression changes with presumably high biological significance, the list of differentially expressed probes, all with a biphasic pattern of regulation from T0 through T2 and T24, was reduced to 38 candidates (Table 4). Within this panel, two genes had duplicate array probes, whereas no reference sequence could be identified for three other probes, leaving 33 known genes as transcriptionally regulated by vorinostat following this stringent statistical analysis of the array data.

**Table 4:** Differentially expressed genes in patients' peripheral blood mononuclear cells during 24 hours of vorinostat treatment. ^a^

Accession no.	Gene b	Gene name	T2 *versus* T0 c (log_2_-fold change)	T24 *versus* T2 c (log_2_-fold change)
NM_005627	*SGK1*	serum/glucocorticoid regulated kinase 1	−1.58	1.65
NM_016478	*ZC3HC1*	zinc finger, C3HC-type containing 1	−1.43	1.39
NM_175571	*GIMAP8*	GTPase, IMAP family member 8	−1.23	1.34
NM_206938	*MS4A7*	membrane-spanning 4-domains, subfamily A, member 7	−1.21	1.06
NM_002467	***MYC***	v-myc myelocytomatosis viral oncogene homolog (avian)	−1.20	1.09
NM_001024938	*SLC2A11*	solute carrier family 2 (facilitated glucose transporter), member 11	−1.16	1.17
NM_004843	*IL27RA*	interleukin 27 receptor, alpha	−1.14	1.05
NM_000104	*CYP1B1*	cytochrome P450, family 1, subfamily B, polypeptide 1	−1.13	1.26
NM_207007	*CCL4L2*	chemokine (C-C motif) ligand 4-like 2	−1.04	1.26
NM_014167	*CCDC59*	coiled-coil domain containing 59	−1.02	1.00
NM_005346	*HSPA1B*	heat shock 70kDa protein 1B	1.82	−2.02
NM_153812	*PHF13*	PHD finger protein 13	1.80	−1.95
NM_001564	*ING2*	inhibitor of growth family, member 2	1.59	−1.71
NM_001564	*ING2*	inhibitor of growth family, member 2	1.56	−1.56
NM_001001870	*none*	none	1.42	−1.28
NM_016639	*TNFRSF12A*	tumor necrosis factor receptor superfamily, member 12A	1.40	−1.33
NM_152339	*SPATA2L*	spermatogenesis associated 2-like	1.38	−1.46
NM_025079	*ZC3H12A*	zinc finger CCCH-type containing 12A	1.36	−1.29
NM_015675	***GADD45B***	growth arrest and DNA-damage-inducible, beta	1.30	−1.17
NM_013368	*SERTAD3*	SERTA domain containing 3	1.24	−1.30
NM_004219	*PTTG1*	pituitary tumor-transforming 1	1.23	−1.35
NM_014711	*CP110*	CP110 protein	1.20	−1.21
NM_005341	*ZBTB48*	zinc finger and BTB domain containing 48	1.14	−1.03
NM_000179	***MSH6***	mutS homolog 6 (E. coli)	1.13	−1.23
NM_153358	*ZNF791*	zinc finger protein 791	1.13	−1.07
NM_006494	*ERF*	Ets2 repressor factor	1.12	−1.06
NR_002734	*PTTG3P*	pituitary tumor-transforming 3, pseudogene	1.11	−1.18
NM_016605	*FAM53C*	family with sequence similarity 53, member C	1.07	−1.13
NM_004219	*PTTG1*	pituitary tumor-transforming 1	1.07	−1.13
not available	*none*	transcribed locus Hs.559604	1.07	−1.08
NM_000024	*ADRB2*	adrenergic, beta-2-, receptor, surface	1.07	−1.07
XM_926814	*none*	none	1.05	−1.19
NM_006806	*BTG3*	BTG family, member 3	1.05	−1.04
NM_031212	*SLC25A28*	solute carrier family 25 (mitochondrial iron transporter), member 28	1.05	−1.00
NM_000465	***BARD1***	BRCA1 associated RING domain 1	1.02	−1.23
NM_004083	***DDIT3***	DNA-damage-inducible transcript 3	1.02	−1.08
NM_052901	*SLC25A25*	solute carrier family 25 (mitochondrial carrier; phosphate carrier), member 25	1.02	−1.06
NM_024954	*UBTD1*	ubiquitin domain containing 1	1.01	−1.01

a Log_2_-fold change higher than 1.0; *P*-value less than 0.01.

b Genes in bold: chosen for validation of expression in the study patients' peripheral blood mononuclear cells (PBMC) samples and human colorectal carcinoma xenograft models.

c T0 represents baseline PBMC samples; T2 and T24 represent PBMC samples collected two and 24 hours, respectively, after the patients had received the daily dose of vorinostat.

**Figure 3:**
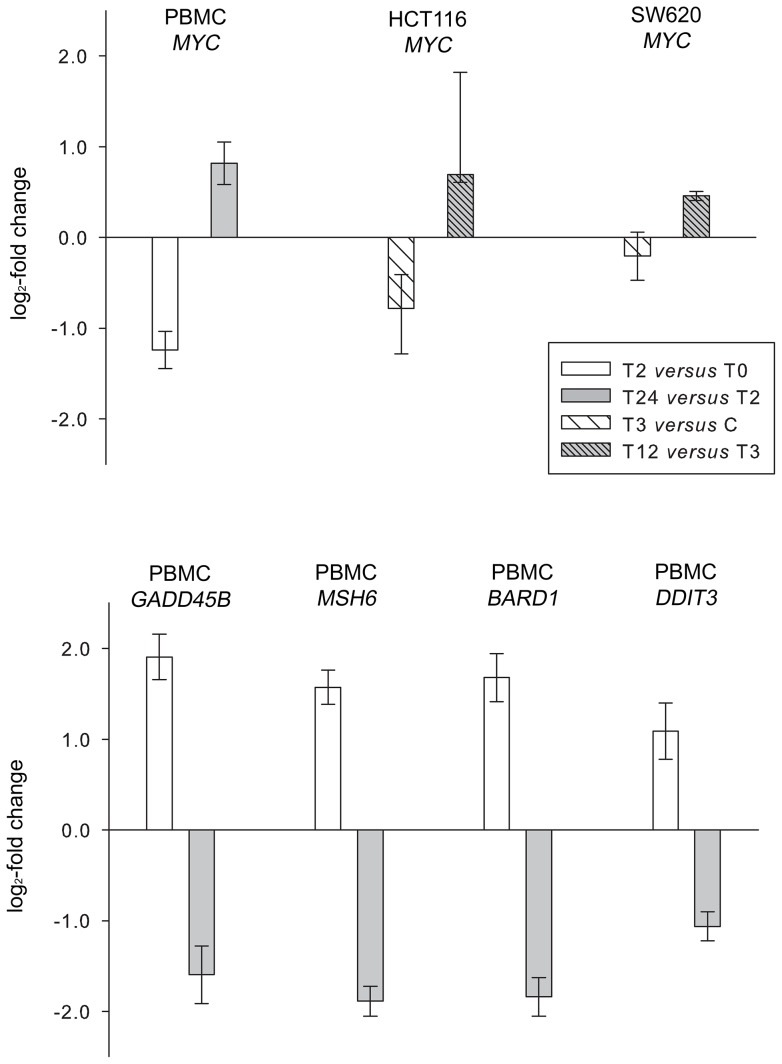
Validation of vorinostat-regulated expression of selected genes. Study patients' peripheral blood mononuclear cells (PBMC) were sampled at baseline (T0) and on-treatment two (T2) and 24 (T24) hours after administration of the daily dose of the study medication vorinostat, and expression of *MYC*, *GADD45B*, *MSH6*, *BARD1*, and *DDIT3* was analyzed by reverse transcriptase quantitative polymerase chain reaction (RT-qPCR). Correspondingly, mice bearing HCT116 or SW620 xenografts were injected intraperitoneally with vehicle (control, C) or vorinostat, and xenografts were harvested three (T3) and 12 (T12) hours after injection for RT-qPCR analysis of *MYC* expression. Relative gene expression (log_2_-fold change) for each comparison is given as mean ± SEM of the PBMC sample values (*n* = 14) and as median and range of the values from control (*n* = 8 for HCT116; *n* = 4 for SW620) and vorinostat-treated (*n* = 4 for HCT116; *n* = 2 for SW620) xenografts. The compared gene expression levels were significantly different within the PBMC (*P*<0.01) and HCT116 (*P*<0.05) sample groups, while the differences were non-significant for the SW620 tumors.

Selection of genes for verification analysis by RT-qPCR was based on both the relevance in the DNA damage response, which is recognized as a significant mechanism contributing to clinical radiation sensitivity [19], and previous indication of regulation by HDAC inhibitors. Five of the 33 genes were found to fulfill both criteria: *MYC* [20], [21] among the ten genes repressed at T2 and correspondingly, *GADD45B* [22], *MSH6* [23], [24], *BARD1* [25], [26], and *DDIT3* [27], [28] among the 23 induced genes; mean PBMC expression levels at T0 relative to reference cell line expression are given in Table S5. These genes were present within the enriched biological processes and pathways identified by the functional annotation analysis of the differentially expressed genes (Table 2 and Table 3), and the biphasic pattern of regulation in PBMC through T2 and T24 was confirmed with significant time-dependent changes (*P*<0.01) for all of the five genes (Figure 3).

### Vorinostat Activity in Experimental Tumors – Validation of Selected Biomarkers

We have previously shown histone hyperacetylation in vorinostat-treated human colorectal carcinoma xenograft models (HCT116 and SW620), peaking three hours after vorinostat administration and with restored baseline levels of histone acetylation three to six hours later, without accumulative effect following repeat daily administration [8]. Hence, expression of the five selected genes was further assessed by RT-qPCR in HCT116 and SW620 xenografts, three and 12 hours after administering vorinostat to tumor-bearing mice; median control expression levels relative to reference cell line expression are given in Table S5. In the HCT116 model, a significant change (*P*<0.05) in vorinostat-induced expression was found for *MYC* only. A similar transient *MYC* repression, but without statistically significant differences in expression levels through the time points, was seen in the SW620 tumors (Figure 3).

### LARC – Primary Tumor *MYC* Expression

On identifying *MYC* repression as a possible biomarker of HDAC inhibitor activity from the strategy of analyzing, firstly, PRAVO study patients' PBMC, and secondly, vorinostat-treated colorectal carcinoma xenografts, and additionally recognizing this drug as a rational approach for biological optimization of radiation effect in pelvic gastrointestinal carcinoma [10], we investigated whether *MYC* might be expressed in the target tissue of a well-established pelvic radiotherapy protocol. In 27 LARC patients receiving neoadjuvant chemoradiotherapy [18], *MYC* expression was detected in all primary tumor samples, though at highly variable levels (median expression value was 0.47 (range 0.020–4.9) relative to reference cell line expression), but was essentially not associated with patient characteristics or treatment outcome in this small cohort (Table S2).

## Discussion

Within the design of the PRAVO phase 1 study (Figure 1), combining the HDAC inhibitor vorinostat with fractionated radiation to pelvic targets volumes for determination of treatment tolerability and response, gene expression array analysis was performed of study patients' PBMC, sampled at baseline (T0) and on-treatment two and 24 hours (T2 and T24) after the patient had received the daily dose of vorinostat, in order to identify possible biomarkers of HDAC inhibitor activity. This strategy revealed 1,600 array probes with biphasic pattern of expression from T0 through T2 and T24 across all of the study patients. A significant number of these genes were found implicated in processes comprising gene regulation, the cell cycle, and chromatin biology. Applying stringent criteria for array data analysis, five genes were recognized both as players in the DNA damage response and targets for regulation by HDAC inhibitors, and were therefore selected for validation of expression pattern both in study patients' PBMC and in human colorectal carcinoma xenograft models. Of these, only *MYC* consistently showed rapid and transient repression in all conditions that were tested.

In the setting of fractionated radiotherapy, a synergistic drug should preferably elicit a radiosensitizing molecular event at each radiation fraction; hence, a pharmacodynamic biomarker should reflect the timing of drug administration with regard to radiation exposure in a periodic manner [1]. Importantly, in a prior preclinical *in vivo* study combining vorinostat and fractionated radiation, we observed that tumor histone acetylation, considered a biomarker of vorinostat activity in the radiotherapy target tissue, reached a maximum three hours after intraperitoneal vorinostat injection into the experimental animals and was restored to baseline acetylation level three to six hours later, but with a repetitive, transient induction of acetylation following repeat injections. Of note, tumor growth inhibition after fractionated radiation, representing a long-term phenotypic outcome of the experimental manipulations, was significantly enhanced both when radiation was delivered at peak and restored histone acetylation levels [8]. Consequently, tumor histone hyperacetylation did not seem to be required at the time of radiation exposure, leaving the question of the optimum temporal relationship between administration of the radiosensitizing drug and radiation delivery unaddressed.

In the PRAVO study, one patient at each vorinostat dose level had both baseline (before commencement of study treatment) and repeat tumor biopsy two-and-a-half hours after administration of vorinostat (on day 3 of the treatment protocol). Histone hyperacetylation was observed in all on-treatment biopsy samples [10], confirming the presence of vorinostat in the target at the time of the daily radiation exposure. However, given that one of the objectives of the study was to determine mechanisms of the presumed radiosensitizing action of vorinostat that were not simultaneously manifesting molecular perturbations elicited by the radiation itself, non-irradiated surrogate tissue was collected for the purpose of identifying new biomarkers. Several investigators have demonstrated PBMC histone hyperacetylation on HDAC inhibitor treatment [14], [29], [30]. With these aspects in mind, PBMC were deemed to represent a relevant surrogate tissue for studying radiosensitizing effects of vorinostat in the context of this clinical trial.

Interestingly, using the study patients' PBMC as surrogate tissue for vorinostat exposure, all of the 1,600 probes that were found to be common for the comparisons T2 *versus* T0 and T24 *versus* T2 in principle represented pharmacodynamic biomarkers of the chosen timing of vorinostat administration in the fractionated radiotherapy protocol. The genes showed rapid and transient induction or repression, thus mirroring the kinetics of the histone acetylation response. This observation implies that the design of the PRAVO study, undertaken in patients with advanced gastrointestinal cancer, may not have provided the optimum context for detailed capture of molecular effects of vorinostat. Thus, ethical concerns may challenge the structure required within a clinical trial setting for evaluating novel biomarker endpoints. Nevertheless, in the PRAVO study, functional annotation analysis of the panel of 1,600 probes identified biological processes and pathways comprising gene regulation (transcription, RNA processing), cell cycle progression (including p53 signaling, commonly involved in the DNA damage response), and chromatin biology. These findings are consistent with well-known cellular perturbations following exposure of experimental tumor models to HDAC inhibitors [2]–[5].

Investigation of biomarkers of HDAC inhibitor activity has been undertaken in a number of clinical therapy trials. These include the demonstration of increased histone acetylation in patients' PBMC in the early trials [14], [29], [30] and the more recent confirmation of changes in tumor expression of acetylated histone and non-histone proteins [10], [14], [31], [32], the HDAC2 enzyme [31] and HR23B protein [33], [34], the latter been proposed as predictive biomarker [35], and of tumor proliferation index [36]. Plasma protein profiling has been done in glioblastoma patients receiving vorinostat in combination with an established cytotoxic regimen [37]. Furthermore, tumor gene expression array analysis has been performed in a study with the HDAC inhibitor panobinostat as single agent [38] and in one trial each of combining either vorinostat or valproate with other biologic agents (in non-small cell lung carcinoma and acute myeloid leukemia, respectively) [39], [40]. To our knowledge, the present study is the first to report on gene expression array analysis as an attempt to identify pharmacodynamic biomarker(s) reflecting timing of HDAC inhibitor administration with regard to an established cytotoxic regimen.

The criteria for selecting genes for validation were both their presumed relevance in the DNA damage response and previous indications of regulation by an HDAC inhibitor [22]–[24], [28], [41], and additionally, in order to find ‘tumor-specific’ markers, omitting genes that typically might be associated with leukocyte biology. Four of the selected genes were induced by vorinostat in the study patients' PBMC but did not show a similar response in the experimental tumor models. *BARD1* encodes a nuclear factor with tumor suppressor activity [24], the stress response effectors encoded by *GADD45B* and *DDIT3* are implicated in cell cycle arrest, DNA repair, and apoptosis [42], [43], and *MSH6* encodes a DNA mismatch repair protein [44]. To date, only three studies seem to have been published on their potential use as biomarkers of therapy response [45]–[47]. In contrast, the confirmation of *MYC* as the only one of the selected genes with rapid and transient change in expression in all tested conditions (*i.e.,* both in the study patients' PBMC and experimental tumor models) may point to a particular importance of myc in the therapeutic setting with fractionated radiation. Future investigations of vorinostat as possible radiosensitizing agent might be within a long-term curative radiotherapy protocol, for example as an additional component of neoadjuvant chemoradiotherapy for LARC. The confirmed presence of *MYC* expression in the intended radiotherapy target tissue (primary rectal tumors) in LARC patients encourages future exploration of this proto-oncogene as a novel biomarker endpoint.

The myc protein acts both as transcriptional activator and repressor, regulating a myriad of genes that collectively conduct cell cycle progression, apoptosis, angiogenesis, and genetic instability [48]. Specifically, it has been suggested that myc activates DNA damage repair genes [20], and interestingly, that myc in hypoxic tumors acts synergistically with the transcription factor hypoxia-inducible factor type 1α, HIF-1α [49], [50]. Recent evidence indicates that HDAC inhibition suppresses HIF-1α activity [51], [52]. Consequently, mitigation of DNA damage repair capacity through suppression of myc/HIF-1α synergy in hypoxic tumors [53], [54], typically being resistant to radiation, provides an appealing explanation for the radiosensitizing effect of HDAC inhibitors.

However, conflicting data have been presented as to how HDAC inhibition may influence the myc protein itself. Whereas inhibition of various HDAC enzymes has been shown to cause myc repression in a range of human cancer cell lines [21], [55]–[57], which corresponds well with the data in the present study, specific nuclear induction of myc to mediate HDAC inhibitor-induced apoptosis in glioblastoma cell lines has also been demonstrated [58]. Interestingly, in nasopharyngeal carcinoma cells that were resistant to radiation, myc was found to be essential through the transcriptional activation of cell cycle checkpoint kinases [59], which are signaling factors implicated in DNA damage repair, thereby facilitating tumor cell survival following radiation exposure. On the contrary, although radiosensitization was conferred by HDAC inhibition both in hypoxic and normoxic hepatocellular carcinoma cells, a lower level of myc expression was associated with the hypoxic and more radioresistant condition [60]. Of particular note, in the present study, the vorinostat-induced repression of *MYC* was found both in study patients' PBMC, clearly representing normoxic tissue, and experimental tumors that also were tested under normoxic conditions.

In conclusion, integral in the PRAVO study design was the collection of non-irradiated surrogate tissue for the identification of biomarker(s) of vorinostat activity to reflect the timing of administration and also suggest the mechanism of action of the HDAC inhibitor. This objective was achieved by gene expression array analysis of study patients' PBMC and as a consequence, the identification of genes that from experimental models are known to be implicated in biological processes and pathways governed by HDAC inhibitors. Importantly, all of the identified genes showed rapid and transient induction or repression and therefore, in principle, fulfilled the requirement of being pharmacodynamic biomarkers for this radiosensitizing drug in fractionated radiotherapy. Among the identified candidate genes, *MYC* repression was found in all patient samples and tested experimental conditions, possibly underscoring the impact of the myc proto-oncogene in this particular therapeutic setting.

## Supporting Information

Table S1 **Short tandem repeat (STR) profiles of cell lines.**
(DOC)

Table S2 **Locally Advanced Rectal Cancer – Radiation Response Prediction (LARC-RRP): patient and treatment characteristics.**
(DOC)

Table S3 **Primers and probes used for reverse transcriptase quantitative polymerase chain reaction analysis.**
(DOC)

Table S4 **Enriched biological processes in patients' peripheral blood mononuclear cells during 24 hours of vorinostat treatment.**
(DOC)

Table S5 **Baseline expression levels of genes assessed by reverse transcriptase quantitative polymerase chain reaction analysis.**
(DOC)
