# Supplementary material for: Biomarkers of Histone Deacetylase Inhibitor Activity in a Phase 1 Combined-Modality Study with Radiotherapy
Source: PLoS One. 2014 Feb 25;9(2):e89750. doi: 10.1371/journal.pone.0089750 (PMC3934935; doi:10.1371/journal.pone.0089750)
Supplement: Table S1 — Short tandem repeat (STR) profiles of cell lines. (DOC) [file pone.0089750.s001.doc]

**Table S1.** Short tandem repeat (STR) profiles of cell lines.

| **Cell name** | **STR designation** | | | | | | | |  |  |  |  |  |  |  |  |  |  |  |
| --- | --- | --- | --- | --- | --- | --- | --- | --- | --- | --- | --- | --- | --- | --- | --- | --- | --- | --- | --- |
|  | **AMEL** | | **CSF1PO** | | **D13S317** | | **D16S539** | | **D5S818** | | | **D7S820** | | **TH01** | | **TPOX** | | **vWA** | |
| HCT116 ATCC ^a^ | x | y | 7 | 10 | 10 | 12 | 11 | 13 | 10 | 11 |  | 11 | 12 | 8 | 9 | 8 | 9 | 17 | 22 |
| HCT116 | x |  | 7 | 11 | 10 | 12 |  | 13 | 10 | 11 |  | 11 | 12 |  |  | 9 | 10 | 17 |  |
| SW620 ATCC ^a^ | x |  | 13 | 14 |  | 12 | 9 | 13 |  |  | 13 | 8 | 9 | 8 |  |  | 11 | 16 |  |
| SW620 | x |  | 13 | 14 |  | 12 | 9 | 13 |  |  | 13 | 8 | 9 | 8 |  |  | 11 | 15 | 16 |

^a^American Type Culture Collection.

The cell lines were subjected to STR analysis for cell line validation using the Promega PowerPlex 16 kit (Promega, Madison, WI, USA), and analyzed on a MegaBace1000 capillary electrophoresis system using Fragment Profiler software (GE Healthcare, Bucks, UK). The STR loci identified were compared to STR profiles published on the ATCC website (http://www.lgcstandards-atcc.org/).
