# Supplementary material for: Biomarkers of Histone Deacetylase Inhibitor Activity in a Phase 1 Combined-Modality Study with Radiotherapy
Source: PLoS One. 2014 Feb 25;9(2):e89750. doi: 10.1371/journal.pone.0089750 (PMC3934935; doi:10.1371/journal.pone.0089750)
Supplement: Table S2 — Locally Advanced Rectal Cancer – Radiation Response Prediction (LARC-RRP): patient and treatment characteristics. (DOC) [file pone.0089750.s002.doc]

**Table S2.** Locally Advanced Rectal Cancer—Radiation Response Prediction (LARC-RRP): patient and treatment characteristics.

|  | ***n* (%)** |
| --- | --- |
| At baseline |  |
| *Median age,* years (range) | 56 (30–70) |
| *Gender* |  |
| Male | 15 (55.6) |
| Female | 12 (44.4) |
| *T-status* |  |
| T3 | 12 (44.4) |
| T4 | 15 (55.6) |
| *N-status* |  |
| N0 | 3 (11.1) |
| N1 | 2 (7.4) |
| N2 | 22 (81.5) |
| *M-status* |  |
| M0 | 23 (85.2) |
| M1 | 4 (14.8) |
| Radiologic response |  |
| *yT-status* |  |
| yT0 | 0 |
| yT1 | 0 |
| yT2 | 1 (3.7) |
| yT3 | 13 (48.1) |
| yT4 | 13 (48.1) |
| *yN-status* |  |
| yN0 | 11 (40.2) |
| yN1 | 4 (14.8) |
| yN2 | 12 (44.4) |
| Histologic response |  |
| *ypT-status* |  |
| ypT0 | 3 (11.1) |
| ypT1 | 0 |
| ypT2 | 3 (11.1) |
| ypT3 | 12 (44.4) |
| ypT4 | 8 (29.6) |
| missing | 1 (3.7) |
| *ypN-status* |  |
| ypN0 | 13 (48.1) |
| ypN1 | 10 (37.0) |
| ypN2 | 3 (11.1) |
| *Tumor Regression Grade* ^a^ |  |
| TRG 1 (ypT0) | 3 (11.1) |
| TRG 2 (sparsely remaining tumor) | 13 (48.1) |
| TRG 3 (intermediate response) | 6 (22.2) |
| TRG 4 (poor response) | 4 (14.8) |
| TRG 5 (poor response) | 0 |
| missing | 1 (3.7) |
| Clinical outcome |  |
| Development of metastatic disease ^b^ | 12 (52.2) |

The LARC-RRP phase 2 study (ClinicalTrials ID NCT00278694) was conducted in patients with locally advanced rectal cancer receiving neoadjuvant chemoradiotherapy (CRT). Data from a subgroup of 27 study patients are presented here. This population was enrolled between July 2008 and March 2010. Patient eligibility criteria, evaluation procedures, study treatment, and review procedures of follow-up have been described in detail previously ^c^. Patients were treated with neoadjuvant fluoropyrimidine-/oxaliplatin-based chemotherapy and CRT, followed by surgery. Follow-up data were obtained from the clinical database and censored on August 31, 2012 ^b^. Valid observations of the presence or absence of distant metastases required designated radiologic examination. Four patients with synchronous resectable liver metastases were excluded from analysis of metastasis-free survival ^b^.

Applying reverse transcriptase quantitative polymerase chain reaction analysis, *MYC* expression was detected in all primary tumor samples, though at highly variable levels (median expression value was 0.47 (range 0.020–4.9) relative to reference LoVo-92 cell line expression). The study cohort was divided into two groups with *MYC* expression levels either lower or higher than the median value, but expression level was essentially not associated with patient characteristics or treatment outcome in this small cohort. The data analysis was performed using Predictive Analytics SoftWare Statistics version 16.0 (SPSS Inc., Chicago, IL, USA).

^a^Histologic Tumor Regression Grade (TRG) of tumor CRT response was graded within one of five TRG categories, spanning from the absence of residual tumor cells in the surgical specimen (histologic complete response; TRG 1) to the lack of morphologic signs of tissue response to treatment (TRG 5).

^b^Censored at a median period of 33 months (range 5–45).

^c^Folkvord S, Flatmark K, Dueland S, de Wijn R, Grøholt KK, et al. (2010) Prediction of response to preoperative chemoradiotherapy in rectal cancer by multiplex kinase activity profiling. Int J Radiat Oncol Biol Phys 78: 555–562.
