# Supplementary material for: Biomarkers of Histone Deacetylase Inhibitor Activity in a Phase 1 Combined-Modality Study with Radiotherapy
Source: PLoS One. 2014 Feb 25;9(2):e89750. doi: 10.1371/journal.pone.0089750 (PMC3934935; doi:10.1371/journal.pone.0089750)
Supplement: Table S3 — Primers and probes used for reverse transcriptase quantitative polymerase chain reaction analysis. (DOC) [file pone.0089750.s003.doc]

**Table S3.** Primers and probes used for reverse transcriptase quantitative polymerase chain reaction analysis.

| **Target** | **Primers ^a^** | **Universal probe** | **NCBI number** | **Amplicon length ^b^** |
| --- | --- | --- | --- | --- |
| *YARS* | F: GGATTAACAGGCAGCAAAATG | # 35 | NM_003680.2 | 67 nt |
|  | R: CCTTCCGATCAAGGAGATCA |  |  |  |
| *TBP* | F: GCTGGCCCATAGTGATCTTT | # 3 | NM_003194.3 | 60 nt |
|  | R: CTTCACACGCCAAGAAACAGT |  |  |  |
| *MYC* | F: CACCAGCAGCGACTCTGA | # 34 | NM_002467.4 | 102 nt |
|  | R: GATCCAGACTCTGACCTTTGC |  |  |  |
| *GADD45B* | F: AGTCGGCCAAGTTGATGAAT | # 34 | NM_015675.2 | 75 nt |
|  | R: CCTCCTCCTCCTCGTCAAT |  |  |  |
| *MSH6* | F: TGAATTGGCAGTTTGTGATGA | # 21 | NM_0000179.2 | 76 nt |
|  | R: TGTTACGTAAGTTGTGCCTACTC |  |  |  |
| *BARD1* | F: ACATTCTGAGAGAGCCTGTGTG | # 88 | NM_000465.2 | 77 nt |
|  | R: TCCAATGCAGTCACTTACACAA |  |  |  |
| *DDIT3* | F: AAGGCACTGAGCGTATCATGT | # 21 | NM_004083.4 | 105 nt |
|  | R: TGAAGATACACTTCCTTCTTGAACAC |  |  |  |

^a^F, forward; R, reverse.

^b^nt, nucleotides.
