# Supplementary material for: Biomarkers of Histone Deacetylase Inhibitor Activity in a Phase 1 Combined-Modality Study with Radiotherapy
Source: PLoS One. 2014 Feb 25;9(2):e89750. doi: 10.1371/journal.pone.0089750 (PMC3934935; doi:10.1371/journal.pone.0089750)
Supplement: Table S4 — Enriched biological processes in patients' peripheral blood mononuclear cells during 24 hours of vorinostat treatment. (DOC) [file pone.0089750.s004.doc]

**Table S4.** Enriched biological processes in patients’ peripheral blood mononuclear cells during 24 hours of vorinostat treatment.

| **Biological process ^a^** | ***n*** | **%** | ***P*-value** |
| --- | --- | --- | --- |
| T2 *versus* T0 ^b^  GO:0006350 transcription | 253 | 17 | 5.1 × 10^-14^ |
| GO:0044265 cellular macromolecule catabolic process | 107 | 7.2 | 8.2 × 10^-10^ |
| GO:0044257 cellular protein catabolic process | 93 | 6.3 | 1.7 × 10^-10^ |
| GO:0007049 cell cycle | 111 | 7.5 | 2.4 × 10^-10^ |
| GO:0051603 proteolysis involved in cellular protein catabolic process | 92 | 6.2 | 2.9 × 10^-10^ |
| GO:0019941 modification-dependent protein catabolic process | 89 | 6.0 | 3.4 × 10^-10^ |
| GO:0043632 modification-dependent macromolecule catabolic process | 89 | 6.0 | 3.4 × 10^-10^ |
| GO:0009057 macromolecule catabolic process | 111 | 7.5 | 3.4 × 10^-10^ |
| GO:0030163 protein catabolic process | 94 | 6.3 | 4.0 × 10^-10^ |
| GO:0006396 RNA processing | 84 | 5.7 | 1.8 × 10^-9^ |
| GO:0045449 regulation of transcription | 276 | 19 | 5.0 × 10^-9^ |
| GO:0022402 cell cycle process | 79 | 5.3 | 3.4 × 10^-7^ |
| GO:0008380 RNA splicing | 48 | 3.2 | 5.4 × 10^-7^ |
| GO:0051276 chromosome organization | 70 | 4.7 | 5.4 × 10^-7^ |
| GO:0006366 transcription from RNA polymerase II promoter | 42 | 2.8 | 6.2 × 10^-7^ |
| GO:0016568 chromatin modification | 46 | 3.1 | 1.2 × 10^-6^ |
| GO:0006351 transcription, DNA-dependent | 48 | 3.2 | 1.2 × 10^-6^ |
| GO:0032774 RNA biosynthetic process | 48 | 3.2 | 1.8 × 10^-6^ |
| GO:0016071 mRNA metabolic process | 56 | 3.8 | 2.0 × 10^-6^ |
| GO:0000278 mitotic cell cycle | 56 | 3.8 | 2.0 × 10^-6^ |
| GO:0006259 DNA metabolic process | 69 | 4.6 | 5.0 × 10^-6^ |
| GO:0006397 mRNA processing | 49 | 3.3 | 7.7 × 10^-6^ |
| GO:0006325 chromatin organization | 55 | 3.7 | 8.3 × 10^-6^ |
| GO:0045184 establishment of protein localization | 93 | 6.3 | 1.6 × 10^-5^ |
| GO:0015031 protein transport | 92 | 6.2 | 1.9 × 10^-5^ |
| GO:0006974 response to DNA damage stimulus | 52 | 3.5 | 4.9 × 10^-5^ |
| GO:0006511 ubiquitin-dependent protein catabolic process | 38 | 2.6 | 5.1 × 10^-5^ |
| GO:0022403 cell cycle phase | 56 | 3.8 | 5.6 × 10^-5^ |
| GO:0008104 protein localization | 101 | 6.8 | 6.1 × 10^-5^ |
| GO:0051726 regulation of cell cycle | 47 | 3.2 | 7.6 × 10^-5^ |
| GO:0033554 cellular response to stress | 69 | 4.6 | 1.8 × 10^-4^ |
| GO:0034660 ncRNA metabolic process | 35 | 2.4 | 1.9 × 10^-4^ |
| GO:0034470 ncRNA processing | 30 | 2.0 | 2.5 × 10^-4^ |
| GO:0010605 negative regulation of macromolecule metabolic process | 84 | 5.7 | 2.8 × 10^-4^ |
| GO:0043933 macromolecular complex subunit organization | 81 | 5.5 | 4.1 × 10^-4^ |
| GO:0010604 positive regulation of macromolecule metabolic process | 94 | 6.3 | 5.0 × 10^-4^ |
| GO:0006281 DNA repair | 39 | 2.6 | 6.7 × 10^-4^ |
| GO:0051301 cell division | 40 | 2.7 | 7.1 × 10^-4^ |
| GO:0048285 organelle fission | 33 | 2.2 | 8.2 × 10^-4^ |
| GO:0051252 regulation of RNA metabolic process | 176 | 11 | 8.8 × 10^-4^ |
| GO:0000279 M phase | 43 | 2.9 | 9.2 × 10^-4^ |
| GO:0065003 macromolecular complex assembly | 75 | 5.1 | 9.5 × 10^-4^ |
| GO:0000087 M phase of mitotic cell cycle | 32 | 2.2 | 0.001 |
| GO:0007067 mitosis | 31 | 2.1 | 0.002 |
| GO:0000280 nuclear division | 31 | 2.1 | 0.002 |
| GO:0016265 death | 79 | 5.3 | 0.002 |
| GO:0006508 proteolysis | 108 | 7.3 | 0.002 |
| GO:0006355 regulation of transcription, DNA-dependent | 170 | 11 | 0.002 |
| GO:0008219 cell death | 78 | 5.3 | 0.002 |
| GO:0070727 cellular macromolecule localization | 49 | 3.3 | 0.003 |
| GO:0045941 positive regulation of transcription | 63 | 4.2 | 0.003 |
| GO:0045934 negative regulation of nucleobase, nucleoside, nucleotide and nucleic acid metabolic process | 58 | 3.9 | 0.003 |
| GO:0045935 positive regulation of nucleobase, nucleoside, nucleotide and nucleic acid metabolic process | 68 | 4.6 | 0.004 |
| GO:0010628 positive regulation of gene expression | 64 | 4.3 | 0.004 |
| GO:0032268 regulation of cellular protein metabolic process | 54 | 3.6 | 0.004 |
| GO:0034613 cellular protein localization | 48 | 3.2 | 0.005 |
| GO:0051186 cofactor metabolic process | 27 | 1.8 | 0.005 |
| GO:0051172 negative regulation of nitrogen compound metabolic process | 58 | 3.9 | 0.005 |
| GO:0051248 negative regulation of protein metabolic process | 26 | 1.8 | 0.005 |
| GO:0012501 programmed cell death | 66 | 4.4 | 0.005 |
| GO:0046907 intracellular transport | 70 | 4.7 | 0.006 |
| GO:0010629 negative regulation of gene expression | 56 | 3.8 | 0.006 |
| GO:0032269 negative regulation of cellular protein metabolic process | 25 | 1.7 | 0.006 |
| GO:0006461 protein complex assembly | 56 | 3.8 | 0.006 |
| GO:0070271 protein complex biogenesis | 56 | 3.8 | 0.006 |
| GO:0051173 positive regulation of nitrogen compound metabolic process | 68 | 4.6 | 0.008 |
| GO:0006915 apoptosis | 64 | 4.3 | 0.009 |
| GO:0016481 negative regulation of transcription | 51 | 3.4 | 0.009 |
| GO:0031328 positive regulation of cellular biosynthetic process | 71 | 4.8 | 0.010 |
| T24 *versus* T2 ^b^  GO:0006350 transcription | 260 | 17 | 8.3 × 10^-16^ |
| GO:0007049 cell cycle | 114 | 7.6 | 2.6 × 10^-11^ |
| GO:0045449 regulation of transcription | 286 | 19 | 5.4 × 10^-11^ |
| GO:0016568 chromatin modification | 55 | 3.7 | 1.3 × 10^-10^ |
| GO:0006396 RNA processing | 86 | 5.7 | 3.7 × 10^-10^ |
| GO:0044265 cellular macromolecule catabolic process | 104 | 6.9 | 8.8 × 10^-10^ |
| GO:0051276 chromosome organization | 78 | 5.2 | 9.0 × 10^-9^ |
| GO:0022402 cell cycle process | 85 | 5.7 | 4.0 × 10^-9^ |
| GO:0044257 cellular protein catabolic process | 89 | 5.9 | 4.3 × 10^-9^ |
| GO:0009057 macromolecule catabolic process | 107 | 7.1 | 6.4 × 10^-9^ |
| GO:0051603 proteolysis involved in cellular protein catabolic process | 88 | 5.9 | 7.4 × 10^-9^ |
| GO:0019941 modification-dependent protein catabolic process | 85 | 5.7 | 8.8 × 10^-9^ |
| GO:0043632 modification-dependent macromolecule catabolic process | 85 | 5.7 | 8.8 × 10^-9^ |
| GO:0030163 protein catabolic process | 90 | 6.0 | 9.4 × 10^-9^ |
| GO:0006325 chromatin organization | 62 | 4.1 | 3.0 × 10^-8^ |
| GO:0006259 DNA metabolic process | 75 | 5.0 | 7.2 × 10^-8^ |
| GO:0000278 mitotic cell cycle | 57 | 3.8 | 9.7 × 10^-7^ |
| GO:0006351 transcription, DNA-dependent | 48 | 3.2 | 1.3 × 10^-6^ |
| GO:0006366 transcription from RNA polymerase II promoter | 41 | 2.7 | 1.7 × 10^-6^ |
| GO:0032774 RNA biosynthetic process | 48 | 3.2 | 1.9 × 10^-6^ |
| GO:0008380 RNA splicing | 46 | 3.1 | 3.3 × 10^-6^ |
| GO:0016071 mRNA metabolic process | 55 | 3.7 | 4.6 × 10^-6^ |
| GO:0006397 mRNA processing | 48 | 3.2 | 1.7 × 10^-6^ |
| GO:0016570 histone modification | 25 | 1.7 | 1.9 × 10^-6^ |
| GO:0016569 covalent chromatin modification | 25 | 1.7 | 3.3 × 10^-5^ |
| GO:0033554 cellular response to stress | 72 | 4.8 | 3.5 × 10^-5^ |
| GO:0045184 establishment of protein localization | 91 | 6.1 | 4.8 × 10^-5^ |
| GO:0006974 response to DNA damage stimulus | 52 | 3.5 | 5.1 × 10^-5^ |
| GO:0015031 protein transport | 90 | 6.0 | 5.7 × 10^-5^ |
| GO:0006281 DNA repair | 42 | 2.8 | 8.0 × 10^-5^ |
| GO:0043933 macromolecular complex subunit organization | 84 | 5.6 | 9.9 × 10^-5^ |
| GO:0022403 cell cycle phase | 55 | 3.7 | 1.1 × 10^-4^ |
| GO:0007050 cell cycle arrest | 21 | 1.4 | 1.1 × 10^-4^ |
| GO:0006511 ubiquitin-dependent protein catabolic process | 36 | 2.4 | 2.6 × 10^-4^ |
| GO:0034470 ncRNA processing | 30 | 2.0 | 2.6 × 10^-4^ |
| GO:0051252 regulation of RNA metabolic process | 180 | 12 | 2.8 × 10^-4^ |
| GO:0010605 negative regulation of macromolecule metabolic process | 84 | 5.6 | 2.9 × 10^-4^ |
| GO:0051726 regulation of cell cycle | 45 | 3.0 | 3.1 × 10^-4^ |
| GO:0051338 regulation of transferase activity | 49 | 3.3 | 3.3 × 10^-4^ |
| GO:0065003 macromolecular complex assembly | 77 | 5.1 | 3.8 × 10^-4^ |
| GO:0008104 protein localization | 97 | 6.5 | 3.9 × 10^-4^ |
| GO:0045859 regulation of protein kinase activity | 46 | 3.1 | 3.9 × 10^-4^ |
| GO:0048285 organelle fission | 34 | 2.3 | 4.0 × 10^-4^ |
| GO:0034660 ncRNA metabolic process | 34 | 2.3 | 4.4 × 10^-4^ |
| GO:0043549 regulation of kinase activity | 47 | 3.1 | 4.6 × 10^-4^ |
| GO:0006355 regulation of transcription, DNA-dependent | 175 | 11 | 4.8 × 10^-4^ |
| GO:0016265 death | 82 | 5.5 | 4.8 × 10^-4^ |
| GO:0008219 cell death | 81 | 5.4 | 6.3 × 10^-4^ |
| GO:0070727 cellular macromolecule localization | 52 | 3.5 | 6.6 × 10^-4^ |
| GO:0042325 regulation of phosphorylation | 57 | 3.8 | 6.8 × 10^-4^ |
| GO:0051301 cell division | 40 | 2.7 | 7.3 × 10^-4^ |
| GO:0042981 regulation of apoptosis | 88 | 5.9 | 8.7 × 10^-4^ |
| GO:0000280 nuclear division | 32 | 2.1 | 8.8 × 10^-4^ |
| GO:0007067 mitosis | 32 | 2.1 | 8.8 × 10^-4^ |
| GO:0034613 cellular protein localization | 51 | 3.4 | 9.8 × 10^-4^ |
| GO:0051174 regulation of phosphorus metabolic process | 58 | 3.9 | 0.001 |
| GO:0019220 regulation of phosphate metabolic process | 58 | 3.9 | 0.001 |
| GO:0043067 regulation of programmed cell death | 88 | 5.9 | 0.001 |
| GO:0000087 M phase of mitotic cell cycle | 32 | 2.1 | 0.001 |
| GO:0045941 positive regulation of transcription | 65 | 4.3 | 0.001 |
| GO:0010941 regulation of cell death | 88 | 5.9 | 0.001 |
| GO:0045934 negative regulation of nucleobase, nucleoside,  nucleotide and nucleic acid metabolic process | 60 | 4.0 | 0.001 |
| GO:0012501 programmed cell death | 69 | 4.6 | 0.002 |
| GO:0006461 protein complex assembly | 59 | 3.9 | 0.002 |
| GO:0070271 protein complex biogenesis | 59 | 3.9 | 0.002 |
| GO:0006352 transcription initiation | 16 | 1.1 | 0.002 |
| GO:0000279 M phase | 42 | 2.8 | 0.002 |
| GO:0010628 positive regulation of gene expression | 66 | 4.4 | 0.002 |
| GO:0010604 positive regulation of macromolecule metabolic process | 91 | 6.1 | 0.002 |
| GO:0006399 tRNA metabolic process | 20 | 1.3 | 0.002 |
| GO:0006473 protein amino acid acetylation | 12 | 0.8 | 0.002 |
| GO:0051172 negative regulation of nitrogen compound metabolic process | 60 | 4.0 | 0.002 |
| GO:0008033 tRNA processing | 15 | 1.0 | 0.002 |
| GO:0006886 intracellular protein transport | 46 | 3.1 | 0.002 |
| GO:0043065 positive regulation of apoptosis | 51 | 3.4 | 0.003 |
| GO:0034621 cellular macromolecular complex subunit organization | 44 | 2.9 | 0.003 |
| GO:0045935 positive regulation of nucleobase, nucleoside, nucleotide  and nucleic acid metabolic process | 69 | 4.6 | 0.003 |
| GO:0006915 apoptosis | 67 | 4.5 | 0.003 |
| GO:0043068 positive regulation of programmed cell death | 51 | 3.4 | 0.003 |
| GO:0051188 cofactor biosynthetic process | 17 | 1.1 | 0.003 |
| GO:0010942 positive regulation of cell death | 51 | 3.4 | 0.003 |
| GO:0006368 RNA elongation from RNA polymerase II promoter | 11 | 0.7 | 0.003 |
| GO:0046907 intracellular transport | 71 | 4.7 | 0.004 |
| GO:0000394 RNA splicing, via endonucleolytic cleavage and ligation | 4 | 0.3 | 0.004 |
| GO:0006388 tRNA splicing, via endonucleolytic cleavage and ligation | 4 | 0.3 | 0.004 |
| GO:0031327 negative regulation of cellular biosynthetic process | 62 | 4.1 | 0.004 |
| GO:0009890 negative regulation of biosynthetic process | 63 | 4.2 | 0.005 |
| GO:0031400 negative regulation of protein modification process | 19 | 1.3 | 0.005 |
| GO:0051186 cofactor metabolic process | 27 | 1.8 | 0.005 |
| GO:0006916 anti-apoptosis | 28 | 1.9 | 0.005 |
| GO:0006354 RNA elongation | 11 | 0.7 | 0.005 |
| GO:0051173 positive regulation of nitrogen compound metabolic process | 69 | 4.6 | 0.005 |
| GO:0010557 positive regulation of macromolecule biosynthetic process | 70 | 4.7 | 0.005 |
| GO:0032259 methylation | 14 | 0.9 | 0.005 |
| GO:0006367 transcription initiation from RNA polymerase II promoter | 13 | 0.9 | 0.006 |
| GO:0016481 negative regulation of transcription | 52 | 3.5 | 0.006 |
| GO:0043543 protein amino acid acylation | 12 | 0.8 | 0.006 |
| GO:0010558 negative regulation of macromolecule biosynthetic process | 60 | 4.0 | 0.006 |
| GO:0022613 ribonucleoprotein complex biogenesis | 25 | 1.7 | 0.006 |
| GO:0006732 coenzyme metabolic process | 22 | 1.5 | 0.008 |
| GO:0042791 5S class rRNA transcription | 4 | 0.3 | 0.008 |
| GO:0042797 tRNA transcription from RNA polymerase III promoter | 4 | 0.3 | 0.008 |
| GO:0000079 regulation of cyclin-dependent protein kinase activity | 11 | 0.7 | 0.008 |
| GO:0010629 negative regulation of gene expression | 55 | 3.7 | 0.009 |
| GO:0006412 translation | 39 | 2.6 | 0.010 |
| GO:0009891 positive regulation of biosynthetic process | 72 | 4.8 | 0.010 |

^a^Gene Ontology (GO) terms.

^b^T0 represents baseline peripheral blood mononuclear cells (PBMC) samples; T2 and T24 represent PBMC samples collected two and 24 hours, respectively, after the patients had received the daily dose of vorinostat.
