# Supplementary material for: Biomarkers of Histone Deacetylase Inhibitor Activity in a Phase 1 Combined-Modality Study with Radiotherapy
Source: PLoS One. 2014 Feb 25;9(2):e89750. doi: 10.1371/journal.pone.0089750 (PMC3934935; doi:10.1371/journal.pone.0089750)
Supplement: Table S5 — Baseline expression levels of genes assessed by reverse transcriptase quantitative polymerase chain reaction analysis. (DOC) [file pone.0089750.s005.doc]

**Table S5.** Baseline expression levels of genes assessed by reverse transcriptase quantitative polymerase chain reaction analysis.

|  | ***MYC*** | ***GADD45B*** | ***MSH6*** | ***BARD1*** | ***DDIT3*** |
| --- | --- | --- | --- | --- | --- |
| Study patients’ PBMC ^a^ | 1.50 ± 0.20 | 6.83 ± 1.0 | 0.35 ± 0.06 | 0.48 ± 0.07 | 0.20 ± 0.03 |
| HCT116 xenograft | 2.12 (0.97–3.3) | 0.75 (0.25–1.9) | 0.69 (0.22–0.86) | 0.45 (0.33–1.6) | 0.19 (0.09–0.35) |
| SW620 xenograft | 3.01 (2.4–4.9) | 1.71 (0.97–2.1) | 1.05 (0.93–1.4) | 0.49 (0.37–0.54) | 0.29 (0.17–0.39) |

^a^Peripheral blood mononuclear cells.

Gene expression levels relative to corresponding expression levels in the LoVo-92 cell line are given; for study patients’ PBMC (*n* = 14), the baseline levels (before commencement of study treatment) as mean ± SEM; for the human HCT116 (*n* = 8) and SW620 (*n* = 4) colorectal carcinoma xenografts, the levels in tumors treated with vehicle (dimethyl sulfoxide) as median and range.
